# Mechanical Tensile Response of Ni–Graphene Nanocomposites in Hydrogen-Irradiation-Coupled Environments Using Molecular Dynamics Simulations

**DOI:** 10.3390/nano15130970

**Published:** 2025-06-22

**Authors:** Tonghe Liu, Xiaoting Yuan, Hai Huang

**Affiliations:** 1 Key Laboratory of Material Physics, Ministry of Education, School of Physics, Zhengzhou University, Zhengzhou 450001, China; 2 International Joint Laboratory for Integrated Circuits Design and Application, Ministry of Education, School of Physics, Zhengzhou University, Zhengzhou 450001, China

**Keywords:** Ni–graphene nanocomposites, mechanical response, hydrogen exposure, displacement damage, radiation tolerance

## Abstract

In Gen-IV nuclear reactors, structural materials must endure unprecedented levels of neutron irradiation and hydrogen exposure, posing significant challenges for traditional Ni-based alloys. This study evaluates Ni–graphene nanocomposites (NGNCs) as a promising solution, leveraging their inherent radiation tolerance and hydrogen diffusion suppression. Using molecular dynamics simulations, we investigate how Ni/graphene interfaces influence mechanical properties under combined hydrogen permeation and displacement damage. Key parameters, such as hydrogen concentration, displacement damage level, strain rate, and temperature, are systematically varied to assess their impact on stress–strain behavior (including Young’s modulus and tensile strength), with comparisons to single-crystal nickel. Our findings reveal that NGNCs exhibit distinct mechanical responses characterized by serrated stress–strain curves due to interfacial slip. Hydrogen and irradiation effects are complex: low hydrogen levels can increase Young’s modulus, while higher concentrations and irradiation generally degrade strength, with NGNCs being more affected than single-crystal nickel. Additionally, NGNCs show enhanced thermal stability but increased strain rate sensitivity. These results provide critical insights for designing materials that balance reinforcement with environmental resilience in nuclear applications.

## 1. Introduction

Designing structural materials with tailored interfaces to effectively mitigate both displacement damage and transmutation gas-induced damage (e.g., hydrogen, helium) has emerged as a predominant research focus in nuclear material development [1,2,3]. Graphene (Gr), distinguished by its remarkable mechanical strength, expansive specific surface area, and minimal weight, exhibits substantial potential for developing highly durable interfaces with metals [4,5]. This approach facilitates the inclusion of numerous metal/Gr interfaces, potentially improving the irradiation tolerance of metallic matrices. A series of recent studies have substantiated this radiation resistance through diverse evidence [6,7,8,9,10,11,12,13]. For example, Yang et al. [7] employed in situ Kr^++^ ion irradiation within transmission electron microscopy to reveal the Cu/Gr interface’s enhanced defect annihilation capability, stemming from its highly extensive stress field distribution. He et al. [8] observed that W–Gr nanocomposites, after 28.92 dpa irradiation, showed a lower hardening rate than pure tungsten. Furthermore, our integrated experimental and computational analyses revealed that Ni/Gr interfaces (NGIs) function as effective defect sinks capable of capturing, absorbing, and annihilating various irradiation-induced defects, including interstitials, vacancies, and hydrogen/helium clusters [9,10,11,12,13].

The advancement of Gen-IV nuclear reactor systems imposes unprecedented demands on structural material performance under extreme operational conditions [14]. Ni-based alloys, renowned for their exceptional elevated-temperature strength and corrosion resistance, have emerged as prime candidates for critical components (e.g., primary circuit vessels and heat exchangers) in molten salt and gas-cooled reactor systems [15,16,17]. Nevertheless, these alloys face substantially higher neutron irradiation levels in Gen-IV reactors compared to those in existing commercial reactors [18,19,20]. This increased exposure generates a multitude of point defects, which, over time, coalesce into defect clusters, including voids and dislocation loops, within the material [21,22,23,24,25,26,27]. Furthermore, over extended operational durations, these alloys are unavoidably subjected to hydrogen-rich conditions [28,29]. For example, in molten salt reactors, significant quantities of hydrogen or tritium atoms are formed through nuclear transmutation reactions in Ni-alloy components [29]. These hydrogen species can penetrate or become embedded in the metallic nickel, subsequently concentrating within irradiation-induced defects [30]. This process elevates hydrogen levels and fosters the creation of hydrogen-stabilized defect clusters [9,31]. Consequently, the interaction between these irradiation-induced defects and hydrogen may trigger the appearance of surface blisters and cracks, thereby hastening hydrogen embrittlement fractures in Ni-based alloys [32,33]. This sequence of processes ultimately undermines the long-term dependability of these materials during operation. As traditional Ni-based alloys rarely endure the extreme conditions of Gen-IV reactors, novel material design approaches are critical for countering the combined challenges of hydrogen and irradiation. Recent breakthroughs in interface engineering suggest potential solutions: Ni–Gr nanocomposites (NGNCs) demonstrate inherent radiation tolerance through Gr-mediated defect recombination and hydrogen diffusion suppression, offering promising pathways for next-generation nuclear structural materials [9,10,11,12,13].

From the perspective of engineering applications, NGNCs must exhibit reliable mechanical stability and consistent performance in settings where hydrogen and irradiation effects are interlinked to be viable for Gen-IV nuclear reactors. However, the scarcity of research in this field compels dependence on analogous studies of metallic nickel for preliminary inferences, which inadequately elucidate the structural evolution and performance dynamics of the composites under such dual hydrogen-irradiation conditions. Furthermore, prevailing experimental methodologies prove inadequate in directly observing the intricate behaviors stemming from these intertwined hydrogen-irradiation effects on the composites, nor can they effectively monitor real-time shifts in mechanical attributes or illuminate the core physical mechanisms involved, owing to the phenomena spanning diverse temporal and spatial scales, ranging from atomic to macroscopic dimensions [34]. In response to these experimental shortcomings, particularly pronounced at the nanoscale and atomic levels, classical molecular dynamics (MD) stands out as an exceptionally apt method, offering precise atomic-scale revelations into the compromised microstructure across picosecond and nanometer ranges, thereby facilitating the exploration of the combined hydrogen-irradiation impacts on the mechanical behavior of materials [35,36]. As a result, this computational approach facilitates systematic investigation of hydrogen-irradiation co-effects on the mechanical responses of NGNCs, thereby guiding future experimental designs and accelerating NGNC implementation in advanced nuclear systems.

Given the pivotal influence of NGIs on the structural integrity and performance of NGNCs, in this study, a sequence of MD simulations was executed to assess how these interfaces govern the mechanical responses under simultaneous hydrogen permeation and displacement damage. Especially critical variables, such as hydrogen concentration, displacement damage level, strain rate, and thermal conditions, are systematically examined to delineate their effects on the mechanical responses of NGNC. Building on the prior characterization of defect evolution in hydrogen-irradiation environments [9], which established foundational insights into microstructural dynamics, this current work focuses on bridging these defect mechanisms to macroscopic mechanical responses. These findings deepen our insights into the load-bearing efficacy of NGNCs containing pre-existing hydrogen and lattice defects while furnishing design principles for engineering irradiation-tolerant nanocomposites in Gen-IV nuclear reactors, where concurrent hydrogen and radiation damage presents formidable material degradation challenges.

## 2. Simulation Methodology

The MD simulations were performed using the LAMMPS (version 3Nov2022) software package [37]. Interactions among Ni atoms were modeled with the embedded atom method (EAM) potential, as developed by Bonny et al. [38]. The adaptive intermolecular reactive empirical bond order (AIREBO) potential was applied to characterize the bonds between C atoms and between C and H atoms [39]. The Lennard–Jones potential was employed to represent the interactions between Ni and C atoms [12]. For interactions involving Ni–H and H–H pairs, the Beck potential was adopted, offering a precise depiction of hydrogen’s energetics and kinetics in nickel, as supported by the findings of Torres et al. [40]. To simulate displacement cascades, all aforementioned potentials were adjusted to include the Ziegler–Biersack–Littmark (ZBL) potential, tailored for short-range effects [41]. 

**Figure 1:**
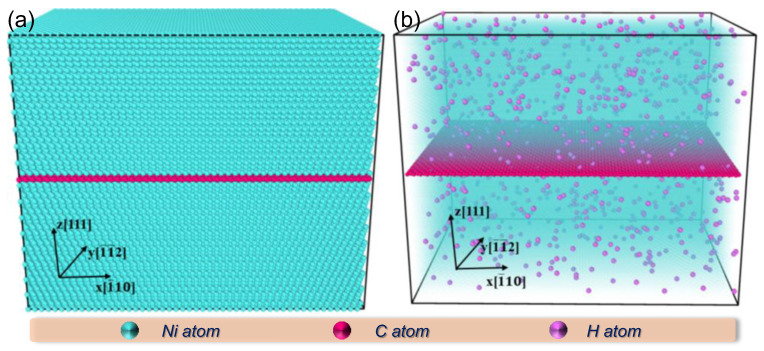
Computational model representations. (**a**) Initial atomic arrangement of the defect-free NGNC. (**b**) Atomic configuration of the NGNC system containing 10,000 appm of hydrogen.

This study initially constructed a sandwich-structured NGI model featuring a *top*–*fcc* configuration, with detailed generation and optimization procedures provided in our prior publications [12,13]. The model’s coordinate system is oriented such that the x-, y-, and z-axes correspond to the [$\bar{1}$10], [$\bar{1}$$\bar{1}$2], and [111] crystallographic directions, respectively. The system encompasses 104,000 atoms, comprising 100,000 Ni atoms and 4000 C atoms, within a simulation cell of 124.6 × 86.3 × 101.9 Å^3^ (see Figure 1a). H atoms were subsequently incorporated at a designated concentration, as depicted in Figure 1b, using a randomized distribution scheme. To emulate an unbounded structure, periodic boundary conditions were enforced across all three spatial axes. After an energy minimization process, the NGI–H system was stabilized using the Nose–Hoover isobaric-isothermal (NPT) ensemble at zero pressure for 40 ps. Thereafter, displacement cascades were activated in the model by designating a Ni atom, located at a set distance from the interface, as the primary knock-on atom (PKA). This PKA was propelled with kinetic energy that was oriented perpendicularly toward the interface. During these cascade events, the system’s outermost layer, approximately 7.4 Å thick, was temperature-controlled via the Nose–Hoover thermostat (NVT ensemble) to promote heat dissipation, while the inner atoms followed adiabatic trajectories in the NVE ensemble. The cascade collisions within hydrogen-containing systems were modeled over a 60 ps duration to establish a stable defect configuration. After this stabilization phase, uniaxial tensile simulations were further conducted on these systems, employing a constant strain rate approach with a 1 fs timestep. Tensile forces were exerted along the *x*-axis of the supercell under NPT ensemble conditions to sustain thermomechanical equilibrium throughout the high-strain-rate deformation process. Along the loading axis, pressure regulation was deactivated, while lateral stresses were held constant at zero. The simulated systems were strained to a 20% engineering strain to explore the synergistic influence of irradiation and hydrogen on their mechanical responses.

Considering factors such as the hydrogen concentration, displacement damage level, strain rate, and temperature that may influence the mechanical response of NGNC, this study systematically examines four scenarios to establish the key parameters for cascade collision and uniaxial tensile simulations. In Scenario I, irradiation was performed with varying hydrogen concentrations (0, 1000, 5000, 10,000, 20,000, and 30,000 appm) at a fixed PKA energy of 5.0 keV, followed by tensile deformation at a constant strain rate of 2 × 10^−3^ ps^−1^, with both processes maintained at 300 K. Scenario II examines a range of PKA energies (0.5 to 10.0 keV) while keeping the hydrogen concentration steady at 10,000 appm. Post-irradiation tensile testing mirrors the conditions of Scenario I, employing a strain rate of 2 × 10^−3^ ps^−1^ and a temperature of 300 K. Scenario III evaluates the influence of varying strain rates (2 × 10^−3^ to 8 × 10^−3^ ps^−1^) on samples previously irradiated at a PKA energy of 5.0 keV with a hydrogen concentration of 10,000 appm, maintaining a constant temperature of 300 K throughout. In Scenario IV, systems irradiated at 5.0 keV PKA energy and 10,000 appm hydrogen were tensile-tested at 2 × 10^−3^ ps^−1^, with temperatures varied across 100, 300, 500, 700, and 900 K. The selected parameter ranges follow established values from prior research for meaningful comparison [42,43,44,45,46,47]. Notably, the MD simulations employ higher strain rates than experimental tests due to computational constraints, preventing direct quantitative comparison but providing valuable atomistic insights into deformation mechanisms [48,49]. Additionally, MD studies confirm material insensitivity to strain rates below 10^−3^ ps^−1^, justifying our selection of higher rates for comparative analysis [50]. For reference, hydrogen-containing nickel single-crystal models (see Figure S1) were also constructed with identical parameter settings to enable comparative studies with the composites.

## 3. Results and Discussion

To investigate the mechanical behavior and parametric sensitivity of NGNC under combined hydrogen-irradiation environments, tensile stress–strain profiles, Young’s modulus, and tensile strength were systematically evaluated. These properties were benchmarked against single-crystal nickel to isolate the functional influence of NGI. Additionally, isolated displacement damage scenarios were investigated to disentangle the individual contributions of hydrogen and irradiation to mechanical degradation in the composite, with corresponding datasets provided in Supplementary Materials for comparative analysis against the following findings.

### 3.1. Hydrogen Concentration Effects on Mechanical Response

**Figure 2:**
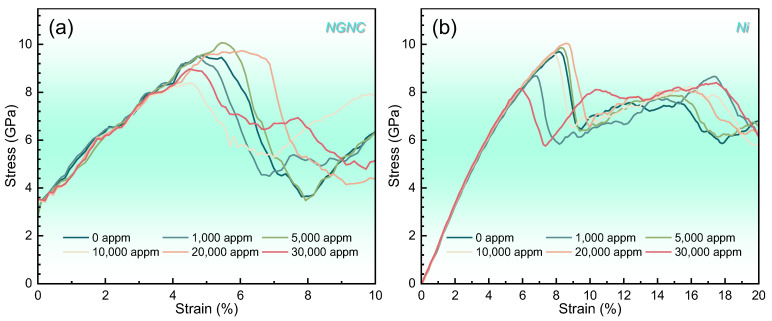
Engineering stress–strain curves measured at 300 K with a PKA energy of 5.0 keV and strain rate of 2 × 10^−3^ ps^−1^, comparing different hydrogen concentrations. (**a**) NGNC. (**b**) Single-crystal nickel.

Figure 2 illustrates the stress–strain behavior of NGNC at 300 K across a range of hydrogen concentrations, compared with that of single-crystal nickel under equivalent conditions. Note that the composite underwent simulated tensile loading up to 20% engineering strain; however, premature fracture occurred prior to reaching 10% strain. Consequently, all mechanical stress–strain curves of NGNC and subsequent analyses focus exclusively on the pre-fracture regime (i.e., strain ≤ 10%). The curves representing NGNC reveal a well-defined elastic phase, a sharp yield point, and a subsequent decline in stress. The presence of hydrogen impurities affects the mechanical properties of the composite more markedly than those of single-crystal nickel. Strikingly, the stress–strain curve of NGNC exhibits a non-zero initial stress attributed to residual internal stresses arising from lattice mismatch at the interface [51,52]. Following structural relaxation, these residual stresses persist, resulting in a pre-stressed state prior to tensile loading. Notably, the NGNC lacks a linear elastic regime, instead displaying pronounced serrated fluctuations during deformation. This nonlinear behavior stems from the weak van der Waals interactions between the Gr layer and nickel matrix, which are orders of magnitude weaker than metallic bonds [53,54]. During loading, stress accumulates until it surpasses the interfacial strength threshold, triggering sudden Gr slippage relative to the nickel matrix. This slip event causes an instantaneous stiffness reduction, marked by abrupt stress drops in the curve. Subsequent re-establishment of interfacial contact restores stiffness, generating the characteristic serrated pattern through cyclic slip-recovery events.

**Figure 3:**
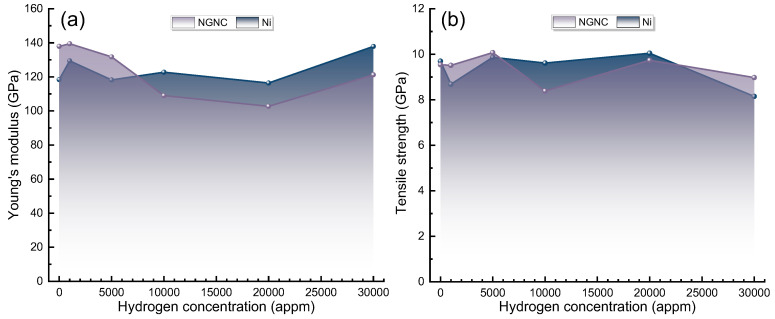
Hydrogen concentration dependence of mechanical parameters for NGNC and single-crystal nickel, measured at 300 K with a PKA energy of 5.0 keV and a strain rate of 2 × 10^−3^ ps^−1^. (**a**) Young’s modulus. (**b**) Ultimate tensile strength.

Figure 3 illustrates the Young’s modulus (E) and ultimate tensile strength (UTS) of NGNC across various hydrogen concentrations, compared to those of single-crystal nickel under equivalent conditions. Note that Young’s modulus, representing the material’s stiffness in the linear elastic regime, was calculated as the ratio of engineering stress (σ_eng) to engineering strain (ε_eng), expressed as E = σ_eng/ε_eng. Ultimate tensile strength, defined as the maximum engineering stress sustained prior to failure, corresponds to the peak value on the stress–strain curve: UTS = max(σ_eng). In Figure 3a, NGNC demonstrates a significantly greater Young’s modulus than single-crystal nickel in the absence of hydrogen doping. Notably, at a hydrogen concentration of approximately 1000 appm, an increase in Young’s modulus is observed in both NGNC and single-crystal nickel, which may be attributed to localized lattice alterations induced by hydrogen at this low concentration [55]. However, with a subsequent rise in hydrogen concentration, Young’s modulus undergoes a decline, likely stemming from the fact that the atomic interactions between hydrogen and nickel are less robust than those forming the metallic bonds [56]. The addition of H atoms into the nickel lattice diminishes the interatomic bonding strength, thereby reducing the structural rigidity. During this phase, the degradation in single-crystal nickel is mitigated by the robustness of its metallic bonds and the restricted solubility of hydrogen [56]. In contrast, NGNC experiences heightened degradation due to Gr’s pronounced attraction to hydrogen, resulting in hydrogen buildup at the interface [57]. This concentrated accumulation of hydrogen exacerbates the weakening of Ni–C interfacial bonds, markedly diminishing the reinforcing impact of Gr and consequently causing a more substantial reduction in NGNC’s Young’s modulus. When hydrogen concentration exceeds 20,000 appm, Young’s modulus shows a recovery, possibly due to structural adaptations or novel interaction mechanisms activated by the elevated hydrogen levels, which partially reinstate the local bonding strength [58]. In a word, the intricate behavior of Young’s modulus is primarily driven by changes in internal atomic interactions and the spatial arrangement of H atoms across varying concentrations. In Figure 3b, as hydrogen concentration progressively increases, the tensile strength undergoes significant variations, underscoring hydrogen’s multifaceted impact on the composite’s microstructure and dislocation dynamics. At a low hydrogen level, a reduction in tensile strength is evident in both NGNC and single-crystal nickel. This decline could be attributed to H atoms inducing localized strain fields and slight lattice imperfections, thereby promoting premature yielding [59]. When hydrogen concentration exceeds 5000 appm, the tensile strength further decreases as hydrogen content rises. Notably, at 20,000 appm, the tensile strength exhibits a resurgence, indicating the potential activation of a novel strengthening mechanism—possibly hydrogen-driven reinforcement or localized structural adjustments—that bolsters resistance to dislocation movement [58]. However, at 30,000 appm, the tensile strength diminishes once more. The surplus of hydrogen likely leads to extensive lattice softening and additional weakening of Ni–C interfacial bonds, thereby impairing the material’s overall mechanical integrity [60].

### 3.2. PKA Energy Effects on Mechanical Response

**Figure 4:**
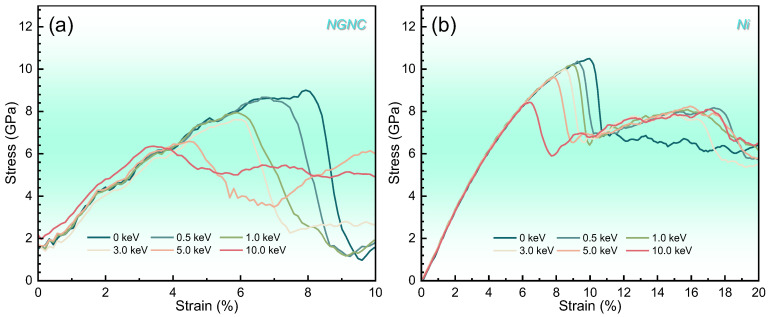
Engineering stress–strain curves measured at 300 K with a hydrogen concentration of 10,000 appm and a strain rate of 2 × 10^−3^ ps^−1^, comparing different PKA energies. (**a**) NGNC. (**b**) Single-crystal nickel.

Figure 4 depicts the stress–strain curves of NGNC across a range of PKA energies from 0.5 to 10 keV, compared with single-crystal nickel under identical irradiation tensile conditions. Every curve reveals a pronounced linear elastic phase, succeeded by a rapid stress decline triggered by fracture. Remarkably, NGNC exhibits steady stress fluctuations within the elastic region, unaffected by varying PKA energies, while single-crystal nickel showcases tensile properties typical of an ideal crystal—evidenced by sharp stress peaks and minimal plastic deformation—suggesting its long-range crystalline structure almost remains intact despite irradiation [44,61]. The consistent serrated elastic behavior observed in NGNC likely arises from sliding at the interface, a process driven predominantly by strain rate (or other external factors) rather than irradiation energy. This interfacial influence overshadows any structural damage caused by irradiation, underscoring the pivotal role of Ni–Gr interactions in shaping mechanical properties. A comparative evaluation indicates that the tensile characteristics of both NGNC and single-crystal nickel gradually diminish with increasing PKA energy, implying that displacement damage governs mechanical deterioration. As evidenced in Figure S1, NGNC exhibits superior strength relative to single-crystal nickel in hydrogen-free and displacement damage-free models; however, incremental increases in displacement damage levels detrimentally affect both materials, with NGNC experiencing disproportionately greater mechanical deterioration. Further analysis of Figure 4 and Figure S2 demonstrates that hydrogen incorporation exacerbates mechanical weakening in both systems. Notably, the coupling of hydrogen and displacement damage induces nonlinear synergistic degradation rather than a simple additive effect—a phenomenon explored in subsequent discussions.

**Figure 5:**
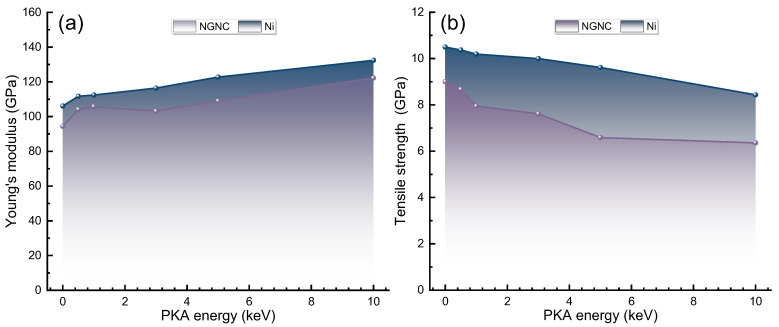
PKA energy dependence of mechanical parameters for NGNC and single-crystal nickel, measured at 300 K with a hydrogen concentration of 10,000 appm and a strain rate of 2 × 10^−3^ ps^−1^. (**a**) Young’s modulus. (**b**) Ultimate tensile strength.

Figure 5 compares Young’s modulus and ultimate tensile strength of NGNC and single-crystal nickel under varying PKA energies. As shown in Figure 5a, both materials exhibit increasing Young’s modulus with rising PKA energy, peaking near 0.5 keV due to irradiation-induced atomic rearrangements that enhance packing density and bonding strength [62]. Moderate PKA energies further amplify modulus increases through dislocation strengthening. Notably, hydrogen-containing systems consistently display lower Young’s modulus than hydrogen-free counterparts (see Figure S3) across all PKA energy levels, with this weakening effect more pronounced in NGNC, suggesting that hydrogen deteriorates mechanical stability under irradiation, particularly in the composite. Figure 5b reveals that the tensile strength of NGNC and single-crystal nickel diminishes progressively as PKA energy rises, exhibiting a trend contrary to that of Young’s modulus. Ma et al. [63] established that high-energy irradiation induces crystallographic disorder, promoting material softening. Irradiation-generated defects fail to impede dislocation motion, precluding irradiation hardening; instead, small-scale defects facilitate dislocation nucleation at reduced stress levels, lowering tensile strength [64,65]. Non-recoverable thermal effects from irradiation further exacerbate softening. In hydrogen-free systems (see Figure S3), unirradiated NGNC exhibits superior tensile strength to single-crystal nickel, confirming Gr’s reinforcement efficacy in pristine conditions. However, NGNC demonstrates heightened irradiation sensitivity: tensile strength declines more rapidly with increasing PKA energy, suggesting Gr transitions from reinforcement to defect aggregation under severe irradiation, diminishing its structural contribution [9]. Hydrogen amplifies this degradation in NGNC, as hydrogen preferentially segregates to the interface, inducing localized stress concentrations and interfacial decohesion that weaken mechanical integrity [9]. On the other hand, hydrogen diffusion may alter the progression of irradiation-generated defects. During irradiation, H atoms could preferentially settle into vacancy sites formed by the process, bonding with them and diminishing the number of unbound vacancies [9]. As vacancies typically diminish tensile strength [66], this hydrogen-vacancy binding may partially offset the reduction in tensile strength, an effect observable in both NGNC and single-crystal nickel systems. These results underscore the competition between Gr’s reinforcement and hydrogen/irradiation-driven degradation, governed by interfacial chemistry and defect dynamics.

### 3.3. Strain Rate Effects on Mechanical Response

**Figure 6:**
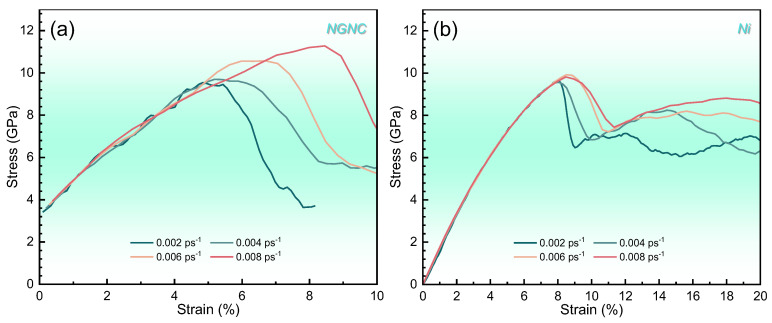
Engineering stress–strain curves measured at 300 K with a PKA energy of 5.0 keV and a hydrogen concentration of 10,000 appm, comparing different strain rates. (**a**) NGNC. (**b**) Single-crystal nickel.

Figure 6 presents the uniaxial tensile stress–strain responses of the NGI models under varying strain rates in comparison with the corresponding single-crystal nickel. Both systems exhibit stress peaks followed by abrupt drops, analogous to fracture initiation in physical materials. A linear elastic regime precedes yield onset, with the NGNC curves displaying pronounced serrated fluctuations within this range, minimized at lower strain rates due to enhanced Ni–Gr interfacial conformity under quasi-static loading. Comparative analysis reveals NGNC’s distinct interface-governed deformation mechanism, characterized by stress redistribution through Gr-mediated load transfer. Notably, increasing strain rates delay yield point attainment in NGNC regardless of hydrogen presence, and hydrogen-free single-crystal nickel shows similar strain rate sensitivity (see Figure S4). In contrast, hydrogen-doped single-crystal nickel exhibits strain-rate-independent yielding, highlighting hydrogen’s role in decoupling the strain rate effects from bulk plasticity. These results underscore Gr’s dual role, i.e., reinforcing stress transfer in pristine conditions while introducing rate-dependent interfacial slip dynamics under deformation.

**Figure 7:**
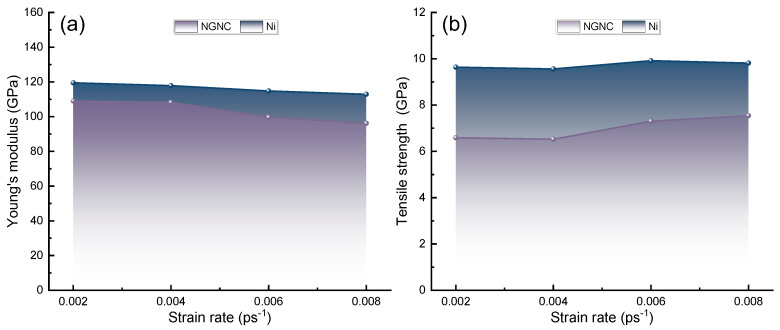
Strain rate dependence of mechanical parameters for NGNC and single-crystal nickel, measured at 300 K with a PKA energy of 5.0 keV and a hydrogen concentration of 10,000 appm. (**a**) Young’s modulus. (**b**) Ultimate tensile strength.

Figure 7 compares the strain-rate-dependent evolution of Young’s modulus and ultimate tensile strength in NGNC and single-crystal nickel. All tested systems demonstrate a consistent inverse relationship between Young’s modulus and tensile strength across strain rates, aligning with the prior observations of strain-rate-mediated mechanical responses [67]. This behavior may arise from strain-rate-dependent deformation mechanisms [68]. Elevated strain rates shorten plastic deformation timescales, limiting dislocation mobility and preventing full strain accommodation, resulting in localized stress accumulation that diminishes Young’s modulus. Concurrently, an incomplete activation of dislocation sources promotes pile-ups, necessitating higher stresses to drive plastic flow, thereby elevating the tensile strength [68]. NGNC exhibits heightened strain rate sensitivity in Young’s modulus relative to single-crystal nickel, with particularly pronounced modulus reductions at elevated rates. This enhanced sensitivity stems from disparate interatomic bonding characteristics—Ni–C interactions differ fundamentally from metallic Ni–Ni bonds, exacerbating energy dissipation challenges at high strain rates and accelerating interfacial slip between Gr and nickel matrix [53,54]. Hydrogen incorporation induces lattice dilatation, increasing interatomic distances and weakening bond strengths. Additionally, hydrogen alters nickel’s coordination environment, replacing metallic Ni–Ni bonds with weaker Ni–H interactions, further compromising structural cohesion and amplifying modulus reductions [60,69]. Notably, hydrogen-free NGNC demonstrates superior tensile strength across all strain rates compared to single-crystal nickel (see Figure S5), showcasing the mechanical benefits of Gr reinforcement. This reinforcement originates from heterointerface-generated barriers to dislocation motion and strain delocalization effects [70]. Hydrogen doping reverses this strengthening trend by degrading Ni–Gr interfacial integrity. Hydrogen accumulation at interfaces reduces bonding efficacy and promotes delamination, significantly diminishing load transfer capacity and tensile strength.

### 3.4. Simulation Temperature Effects on Mechanical Response

**Figure 8:**
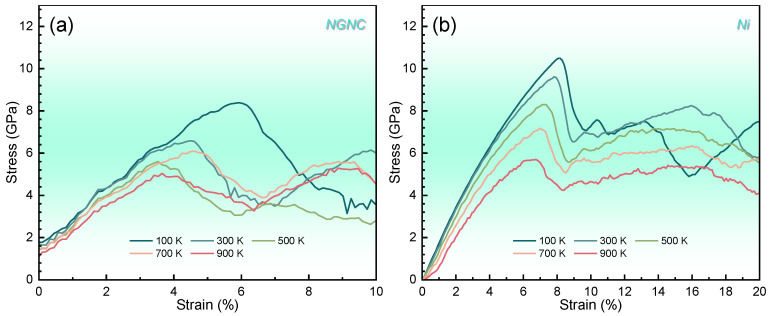
Engineering stress–strain curves measured at a strain rate of 2 × 10^−3^ ps^−1^ with a PKA energy of 5.0 keV and a hydrogen concentration of 10,000 appm, comparing different simulation temperatures. (**a**) NGNC. (**b**) Single-crystal nickel.

During tensile deformation, elevated temperatures induce thermal activation effects that critically govern macroscopic mechanical behavior. Figure 8 compares the uniaxial stress–strain responses of NGNC and single-crystal nickel across varied thermal conditions. Both systems exhibit linear elasticity followed by fracture-induced stress release at peak stress. Combining Figure 8 and Figure S6, a consistent temperature-dependent reduction in yield strength is observed across all systems, unaffected by hydrogen presence, indicating thermal activation of dislocation motion as the dominant yield acceleration mechanism [71]. This trend—characterized by decreasing yield points with rising temperature—reflects enhanced dislocation glide and cross-slip, enabled by thermal energy input, which overcomes lattice friction and accelerates plastic flow initiation. The invariance of this thermal softening behavior to hydrogen doping suggests that temperature-driven dislocation dynamics supersede hydrogen-defect interactions in controlling yield kinetics under thermomechanical loading.

**Figure 9:**
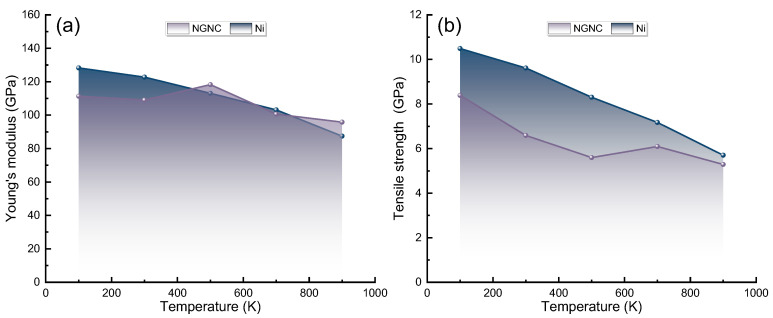
Simulation temperature dependence of mechanical parameters for NGNC and single-crystal nickel, measured at a strain rate of 2 × 10^−3^ ps^−1^ with a PKA energy of 5.0 keV and a hydrogen concentration of 10,000 appm. (**a**) Young’s modulus. (**b**) Ultimate tensile strength.

Figure 9 presents a comparative analysis of the Young’s modulus and ultimate tensile strength between NGNC and single-crystal nickel under identical thermal conditions. As shown in Figure 9a and Figure S7a, the Young’s modulus of single-crystal nickel—both hydrogenated and hydrogen-free—progressively declines with rising temperatures, aligning with established literature [67]. This behavior arises from two primary factors: (1) amplified atomic thermal vibrations at higher temperatures, which expand interatomic spacing and weaken bonding forces, and (2) thermally induced defects, such as vacancies and dislocations, which accumulate significantly and diminish material stiffness [23,72]. Conversely, NGNC displays a distinct non-monotonic modulus response: the values initially rise with temperature, reaching a maximum at 500 K, before decreasing, regardless of the hydrogen content. This deviation originates from Gr’s dual stabilizing roles [73,74,75,76]. First, Gr restricts thermal expansion in the nickel matrix, counteracting lattice distortion. Second, its two-dimensional architecture impedes defect formation and propagation under thermal stress. Furthermore, robust interfacial bonding between Gr and the nickel matrix generates a pinning effect that curtails dislocation movement, thereby preserving mechanical integrity at elevated temperatures [73,74,75,76]. Collectively, these mechanisms grant NGNC enhanced thermal stability relative to its single-crystal counterpart. In Figure 9b and Figure S7b, NGNC’s tensile strength diminishes with temperature due to intensified thermal activation processes. Elevated temperatures facilitate dislocation glide and cross-slip, promoting plastic deformation. In irradiated environments, pre-existing defects and hydrogen species exhibit increased mobility, exacerbating damage through defect clustering and lattice distortion. These microstructural changes ultimately degrade yield strength. However, NGNC demonstrates a markedly lower strength reduction compared to single-crystal nickel, indicating Gr’s capacity to mitigate thermal softening. This resilience likely stems from Gr’s ability to obstruct dislocation motion and stabilize defect configurations, underscoring its utility in high-temperature structural applications [70].

## 4. Conclusions

In summary, this study has employed molecular dynamic simulations to systematically investigate how Ni/graphene interfaces (NGIs) influence the mechanical properties of Ni–graphene nanocomposites (NGNCs) under various conditions, including different hydrogen concentrations, displacement damage levels, strain rates, and temperatures, with direct comparison to single-crystal nickel. These findings reveal that hydrogen and irradiation generally degrade Young’s modulus and tensile strength, though the effects vary. At low hydrogen levels (~1000 appm), Young’s modulus rises due to lattice changes but declines at higher concentrations as hydrogen weakens bonds. Displacement damage, modeled with PKA energies, reduces tensile strength, with NGNC showing greater sensitivity due to Gr’s role in defect clustering under severe irradiation. The strain rate changes highlight NGNC’s unique interfacial behavior, with serrated stress–strain curves reflecting slip at the NGI. The temperature impacts also differ: single-crystal nickel’s modulus decreases steadily with heat, while NGNC peaks at 500 K, benefiting from Gr’s stabilizing effect. Compared to single-crystal nickel, NGNC offers superior strength in pristine states but is more vulnerable to combined hydrogen-irradiation damage. Gr enhances mechanical integrity in ideal conditions but can exacerbate weaknesses when hydrogen accumulates at the interfaces, disrupting bonding and load transfer. These insights reveal Gr’s dual role, i.e., reinforcing and destabilizing, depending on the environmental stressors. This study provides valuable guidance for designing irradiation-tolerant materials for advanced nuclear reactors, emphasizing the critical influence of interfacial dynamics in nanocomposites under extreme conditions.

## Data Availability

The data that support the findings of this study are available from the corresponding author upon reasonable request.

## Supplementary Materials

The following supporting information can be downloaded at: https://www.mdpi.com/article/10.3390/nano15130970/s1, Figure S1: Computational model representations. (a) Initial atomic arrangement of the defect-free single-crystal nickel. (b) Atomic configuration of the single-crystal nickel containing 10,000 appm of hydrogen. Figure S2: Engineering stress–strain curves measured at 300 K with a strain rate of 2 × 10^−3^ ps^−1^ in hydrogen-free environments, comparing different PKA energies. (a) NGNC. (b) Single-crystal nickel; Figure S3: PKA energy dependence of mechanical parameters for NGNC and single-crystal nickel, measured at 300 K with a strain rate of 2 × 10^−3^ ps^−1^ in hydrogen-free environments. (a) Young’s modulus. (b) Ultimate tensile strength; Figure S4: Engineering stress–strain curves measured at 300 K with a PKA energy of 5.0 keV in hydrogen-free environments, comparing different strain rates. (a) NGNC. (b) Single-crystal nickel; Figure S5: Strain rate dependence of mechanical parameters for NGNC and single-crystal nickel, measured at 300 K with a PKA energy of 5.0 keV in hydrogen-free environments. (a) Young’s modulus. (b) Ultimate tensile strength; Figure S6: Engineering stress–strain curves measured at a strain rate of 2 × 10^−3^ ps^−1^ with a PKA energy of 5.0 keV in hydrogen-free environments, comparing different simulation temperatures. (a) NGNC. (b) Single-crystal nickel; Figure S7: Simulation temperature dependence of mechanical parameters for NGNC and single-crystal nickel, measured at a strain rate of 2 × 10^−3^ ps^−1^ with a PKA energy of 5.0 keV in hydrogen-free environments. (a) Young’s modulus. (b) Ultimate tensile strength.
