# Supplementary material for: Mechanical Tensile Response of Ni–Graphene Nanocomposites in Hydrogen-Irradiation-Coupled Environments Using Molecular Dynamics Simulations
Source: Nanomaterials (Basel). 2025 Jun 22;15(13):970. doi: 10.3390/nano15130970 (PMC12250986; doi:10.3390/nano15130970)
Supplement: Supplementary file 1 [file nanomaterials-15-00970-s001.zip › nanomaterials-3660974-supplementary.pdf]

Supplementary Information

Mechanical tensile response of Ni−graphene nanocomposites in hydrogen-irradiation coupled environments using molecular dynamics simulations

Tonghe Liu[a,1], Xiaoting Yuan[a,b,1], Hai Huang[a,*]

[a] *Key Laboratory of Material Physics, Ministry of Education, School of Physics, Zhengzhou University, Zhengzhou 450001, China.*

[b] *International Joint Laboratory for Integrated Circuits Design and Application, Ministry of Education, School of Physics, Zhengzhou University, Zhengzhou 450001, China.*

Total Pages: 4 excluding cover pages

Total Figureures: 7

[*] Corresponding author.
E-mail address: huanghai@zzu.edu.cn (H. Huang).
[1] Authors contributed equally.

1. Supplementary methodology

The coordinate system of single-crystal nickel model is oriented such that the x-, y-, and z-axes correspond to the $[\bar{1}10]$, $[\bar{1}\bar{1}2]$, and [111] crystallographic directions, respectively. The system encompasses 100,000 Ni atoms, within a simulation cell of 124.2 × 86.0 × 101.5 Å^3 (see Figure. S1(a)). H atoms were subsequently incorporated at a designated concentration, as depicted in Figure. S1(b), using a randomized distribution scheme.

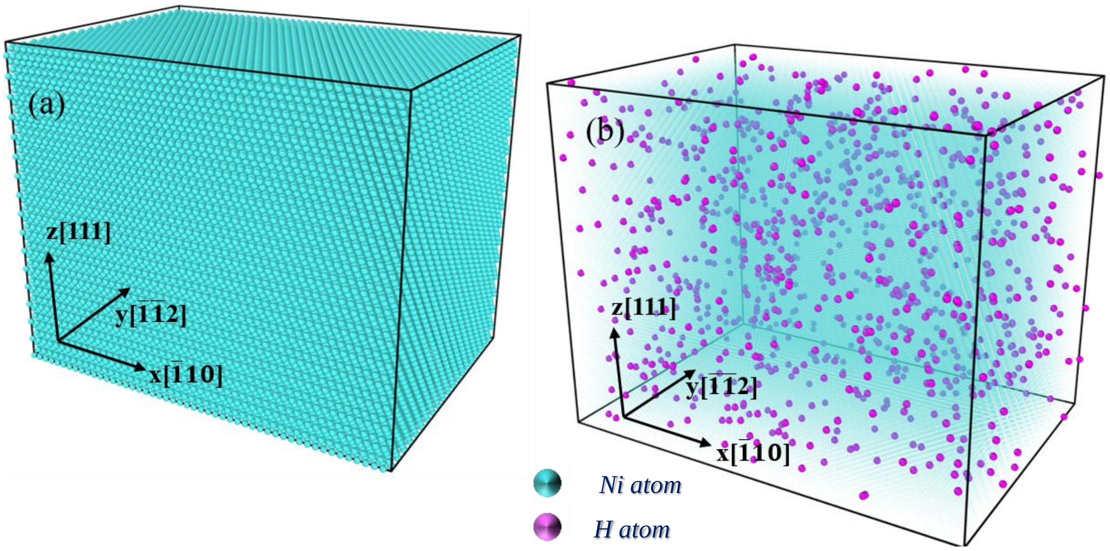

Figure S1. Computational model representations. (a) Initial atomic arrangement of the defect-free single-crystal nickel. (b) Atomic conFigureuration of the single-crystal nickel containing 10,000 appm of hydrogen.

2. Supplementary results

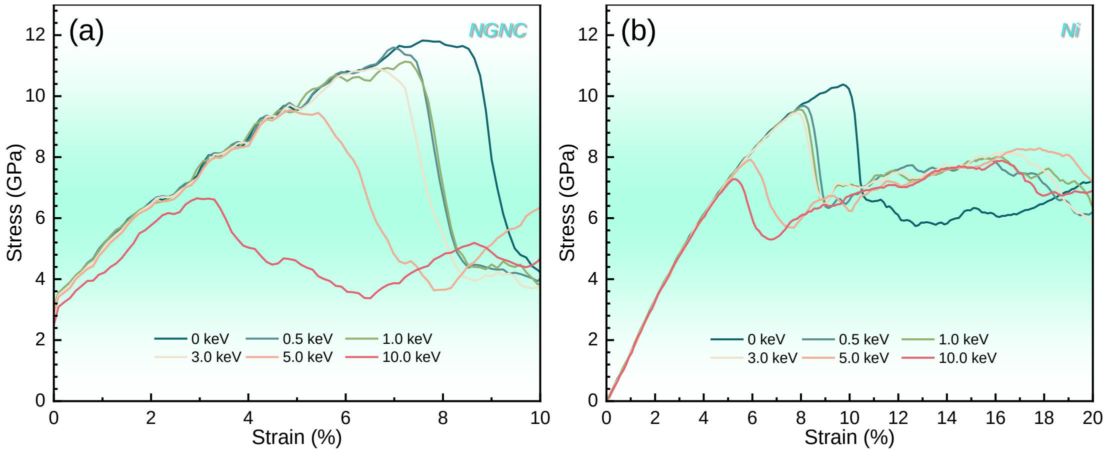

Figure S2. Engineering stress–strain curves measured at 300 K with a strain rate of 2×10^{-3} ps^{-1} in hydrogen-free environments, comparing different PKA energies. (**a**) NGNC. (**b**) Single-crystal nickel.

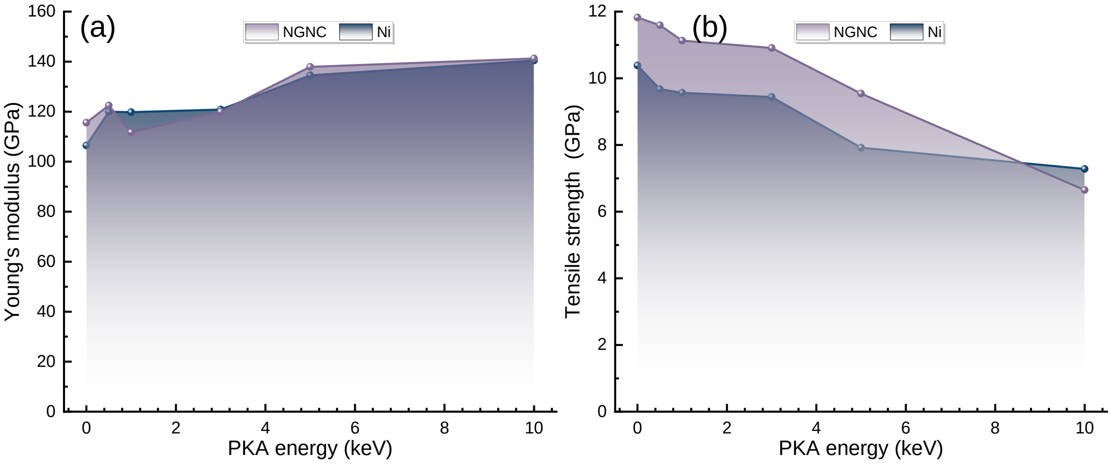

Figure S3. PKA energy dependence of mechanical parameters for NGNC and single-crystal nickel, measured at 300 K with a strain rate of 2×10^{-3} ps^{-1} in hydrogen-free environments. (**a**)Young's modulus. (**b**) Ultimate tensile strength.

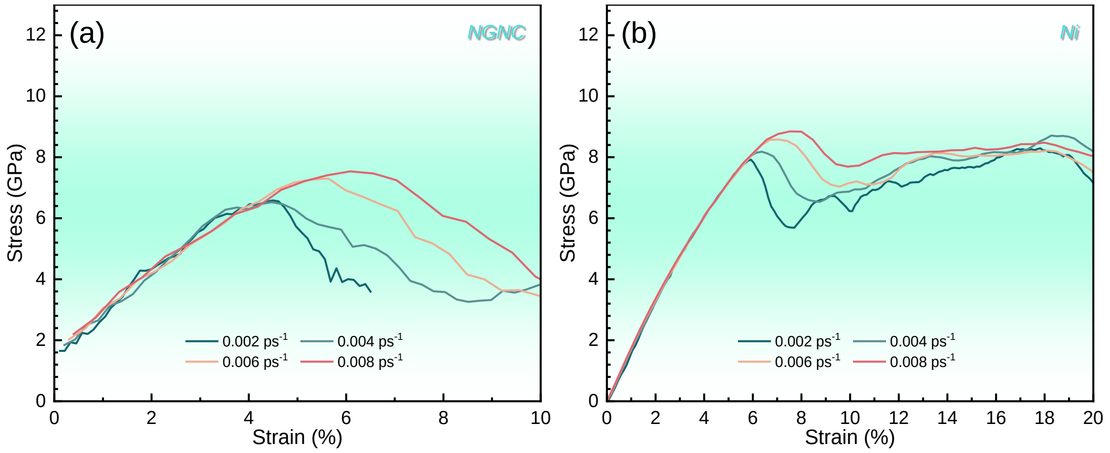

Figure S4. Engineering stress–strain curves measured at 300 K with a PKA energy of 5.0 keV in hydrogen-free environments, comparing different strain rates. (**a**) NGNC. (**b**) Single-crystal nickel.

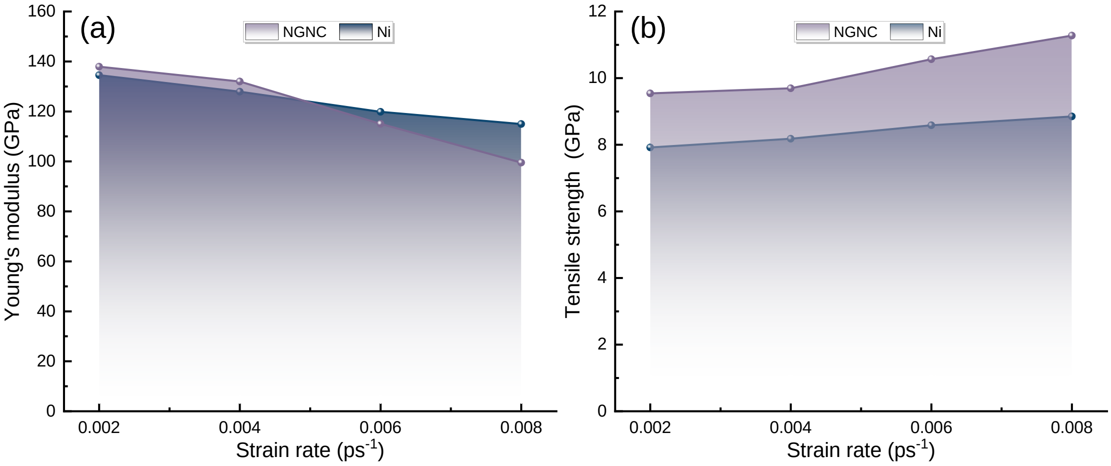

Figure S5. Strain rate dependence of mechanical parameters for NGNC and single-crystal nickel, measured at 300 K with a PKA energy of 5.0 keV in hydrogen-free environments. (**a**)Young's modulus. (**b**) Ultimate tensile strength.

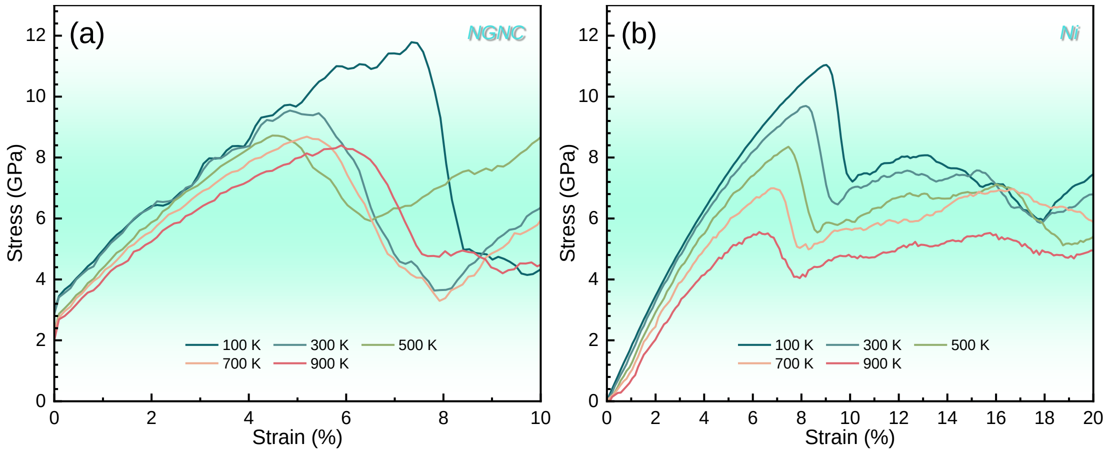

Figure S6. Engineering stress–strain curves measured at a strain rate of 2×10^{-3} ps^{-1} with a PKA energy of 5.0 keV in hydrogen-free environments, comparing different simulation temperatures. (**a**) NGNC. (**b**) Single-crystal nickel.

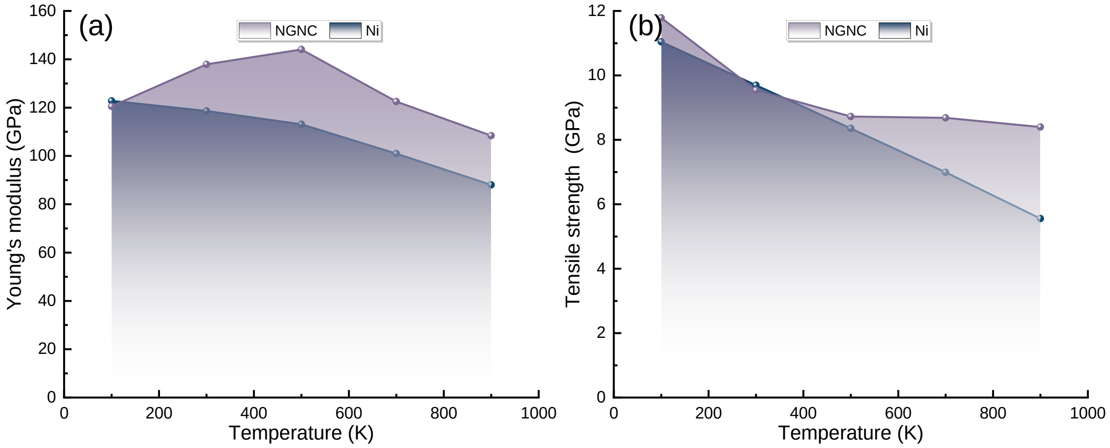

Figure. S7. Simulation temperature dependence of mechanical parameters for NGNC and single-crystal nickel, measured at a strain rate of 2×10^{-3} ps^{-1} with a PKA energy of 5.0 keV in hydrogen-free environments. (**a**)Young's modulus. (**b**) Ultimate tensile strength.
